# The Genome-Wide Study of Human Social Behavior and Its Application in Sociology

**DOI:** 10.3389/fsoc.2019.00053

**Published:** 2019-06-26

**Authors:** Peter T. Tanksley, Ryan T. Motz, Rachel M. Kail, J. C. Barnes, Hexuan Liu

**Affiliations:** ^1^School of Criminal Justice, University of Cincinnati, Cincinnati, OH, United States; ^2^Institute for Interdisciplinary Data Science, University of Cincinnati, Cincinnati, OH, United States

**Keywords:** social science genomics, sociogenomics, genome-wide association, polygenic scores, heritability, candidate genes, gene-environment interaction, gene-environment correlation

## Abstract

Recent years have seen a push for the integration of modern genomic methodologies with sociological inquiry. The inclusion of genomic approaches promises to help address long-standing issues in sociology (e.g., selection effects), as well as open up new avenues for future research. This article reviews the substantive findings of behavior genetic/genomic research, both from the recent past (e.g., twin/adoption studies, candidate gene studies) and from contemporary genomic analyses. The article primarily focuses on modern genomic methods available to sociologists (e.g., polygenic score analysis) and their various applications for answering sociological questions. The article concludes by considering a number of areas to which genomic researchers and sociologists should pay close attention if a consilience between genomic methods and sociological research is to be fully realized.

## Introduction

Social scientists have long been interested in understanding what role biology plays in human social traits and behaviors. With the completion of the Human Genome Project at the turn of the twenty-first century and the subsequent advent of genome-wide data and methods, researchers are addressing this question in unprecedented ways. Genome-wide analyses, and particularly the polygenic score (PGS) analysis, have already made significant contributions in the health and life sciences such as medicine and epidemiology, and even the social sciences such as psychology and economics. The use of genomic methods in sociology, albeit growing, has not yet been fully realized. In other words, we agree with Conley and Fletcher (2017, p. 11) that, “…the social genomics revolution is just getting started.” This review will examine the substantive findings of research that has explored the application of genomic approaches to sociological questions.

The purpose of this article is to review the past and current findings that have bearing on sociogenomics work. We will also offer thoughts and predictions about the future direction(s) of sociogenomics research. Thus, this article begins by providing a brief description of the behavior genetic research that preceded sociogenomics, beginning with twin/adoption studies and candidate gene studies. The bulk of the paper will focus on the contemporary era and, in particular, on genome-wide association (GWA) and PGS studies, with special focus turned toward the substantive findings and possible uses of these methods in sociological research. We will then conclude by considering some of the current and future issues that sociogenomics research will need to address in the coming years.

This article hopes to encourage familiarity with sociogenomic methods among sociological researchers. We also wish to establish the importance of answering a central question in sociogenomics: what role does biology play in social traits and behaviors? On this point, we believe that the eminent biologist E.O. Wilson was remarkably prescient in his book, *Consilience* (Wilson, 1999), in outlining the necessary steps to attaining the answer to that central question of biology and behavior. His words will provide a guide and inspiration for the sections that follow.

**“*The clarification of norms of reaction and heritability*…* is the crucial first step toward unbraiding the roles of heredity and environment in human behavior*…”**

**-**E.O. Wilson, *Consilience* (Wilson, 1999, p. 155–156)

## Background

Some of the earliest work on investigating the role of biology in human social behavior began in the 1900s and used the approach known as “variance partitioning” (Tabery, 2014). The variance partitioning approach evaluates the heritability (i.e., the proportion of variance explained by genetics) of traits or behaviors, typically with the use twin and adoption studies. These family-based studies provided a natural experiment wherein the actual amount of genetic relatedness between individuals could be known (e.g., 50 ad 100% for fraternal and maternal twins, respectively, and 0% for non-biological siblings). Understanding the genetic relatedness of family members allowed researchers to partition the variation in social traits and behaviors in terms of their genetic (i.e., heritability) and environmental contributions.

Overall, twin research supported the hypothesis that genes play a crucial role in the development of human traits and behaviors. This support was strong enough that Turkheimer (2000) set forth three laws of behavior genetics: (1) “all human behavior is heritable,” and (2) “the effect of being raised in the same family is smaller than the effect of genes,” but (3) “a substantial portion of the variation in complex human behavioral traits is not accounted for by the effects of genes or families” (p. 160). These laws concisely and accurately summarized the conclusions of the first era of behavior genetic research. By evaluating over 2,500 publications and over 17,000 traits, Polderman et al. (2015) demonstrated that *all* human traits are heritable. Overall, ~50% of the variation in phenotypes (i.e., observable traits or behaviors) was explained by genetic influences including characteristics such as temperament and conduct disorder. Furthermore, Polderman et al. (2015) indicated that additive genetic influence was the best explanation for 69% of traits, suggesting that twin studies were an effective and efficient method to evaluate the influence of genes on trait variance.

**“*The logical next step is the location of the genes that affect behavior.”***

**-** E.O. Wilson, *Consilience* (Wilson, 1999, p. 155–156)

Toward the end of the first era of behavior genetic research, scholars aimed to go beyond simply addressing *how much* genes matter in behavior (i.e., the variance partitioning approach), and instead sought to answer the deeper questions of *which genes* and *why*—known as the “mechanism elucidation” approach (Tabery, 2014). The second era was launched under the impression that there were only a few genetic mechanisms with large effects that, when combined, would account for much of the heritability of an outcome. Researchers came to use the “candidate gene” approach, wherein they would identify a gene of interest (i.e., the genetic “candidate”) *a priori* and conduct statistical analyses to see if any variants of the gene (i.e., alleles) were associated with the behavioral outcome of interest.

Of particular interest is the phenomenon of gene-environment (G × E) interaction. Simply put, a G × E interaction suggests the effect of the environment depends on genes or, the reverse, that the effect of genes depends on the environment (Shanahan and Hofer, 2005; Rutter, 2006; Boardman et al., 2013). Two of the most groundbreaking (G × E) studies using candidate genes were performed by Caspi et al. (2002, 2003). In the first study (Caspi et al., 2002), the authors investigated the interactive relationship between variants of the *MAOA* gene and childhood maltreatment on antisocial behavior in adulthood. In the second study (Caspi et al., 2003), the authors investigated the interactive relationship between variants in the *5-HTTLPR* gene and stressful life events on depression. In both studies, the authors found that the *MAOA* and the *5-HTTLPR* candidate genes alone did not predict antisocial behavior and depression, respectively. Crucially, however, when the alleles of the candidate genes were interacted with their respective environmental exposures, significant relationships emerged. These findings suggest that some behavioral traits are co-dependent on (1) the possession of genetic risk and (2) exposure to environmental stressors in order for them to manifest.

The enthusiasm for the candidate gene era lost momentum, however, as subsequent studies using the same genetic variants and same outcomes in different samples came to inconsistent conclusions and oftentimes completely failed to replicate (e.g., Chabris et al., 2012). For example, Duncan and Keller (2011) performed a thorough review of the first tens year of G × E studies using candidate genes in psychiatry and found that while 96% of novel findings were significant, they only replicated 27% of the time. A number of reasons have been presented for why candidate gene studies suffered from inconsistent findings and failure to replicate (e.g., see Tabor et al., 2002); however, one of the key failures of the second era of behavior genetic research was the assumption that only a few genetic variants played a role in complex human traits and behaviors.

**“*Once the genes have been mapped on the chromosomes and their pathways of expression identified, their interaction with the environment can be more precisely traced.”***

**-** E.O. Wilson, *Consilience* (Wilson, 1999, p. 155–156)

## Sociogenomics

In the past decade, a new polygenic model has been adopted in the study of complex human traits. The polygenic model assumes complex traits are the result of “…unknown but certainly multifarious, and interactive biological and social pathways, with large numbers of genetic loci” (i.e., “weak biologism”; Turkheimer, 1998, p. 787). By the early 2010s, GWA studies provided support for the polygenic model by locating hundreds of common genetic variants across the genome that bore significant associations with complex traits such as human height, body mass, and educational attainment (Wood et al., 2014; Locke et al., 2015; Lee et al., 2018). In 2015, Chabris et al. published an article wherein they proffered a “fourth law of behavior genetics” to supplement Turkheimer's original three. The fourth law: “A typical human behavioral trait is associated with very many genetic variants, each of which accounts for a very small percentage of the behavioral variability” (Chabris et al., 2015, p. 305).

### Genome-Wide Association Studies

Early GWA studies focused on diseases (e.g., age-related macular degeneration [AMD]) and other medically-relevant physical traits like body mass index and height. Only a short time later, however, researchers were able to conduct GWA studies on complex traits that were of interest to social scientists. Some recent GWA studies have examined traits such as intelligence (Savage et al., 2018), subjective well-being (Okbay et al., 2016a), attention-deficit/hyperactivity disorder (ADHD) (Demontis et al., 2019), aggression (Pappa et al., 2016), antisocial behavior (Tielbeek et al., 2017), tobacco, and alcohol use (Liu et al., 2019), and educational attainment (Rietveld et al., 2013a; Okbay et al., 2016b; Lee et al., 2018).

The study of educational attainment provides a prototypical example for how GWA studies progress in terms of gene discovery and explanatory power. The first GWA study of educational attainment (EA1; Rietveld et al., 2013a) had a sample size of ~126,000 individuals. The study reported finding three genome-wide significant single nucleotide polymorphisms (SNPs) that explained ~2% of the variation in years of education. Three years later, EA2 (Okbay et al., 2016b) boasted a sample of nearly 300,000 individuals and succeeded in discovering 74 significant SNPs that explained ~4% of the variation in education. The most recent study, EA3 (Lee et al., 2018), used a sample of 1.1 million individuals and discovered 1,274 genome-wide significant SNPs that explained 11–13% of the variation in years of education.

Although GWA is a method for gene-discovery, the large number and small effect sizes of the genetic variants identified by GWA studies largely preclude researchers from identifying specific causal pathways. Contrary to the initial assumption that GWA studies would find a small number of highly impactful SNPs, Plomin (2018) writes that “…what [GWA studies] found was gold dust, not nuggets” (p. 187). Though small, when the effects of SNPs are combined, a significant proportion of the variance in complex traits can be predicted. Plomin (2018) continues, “[e]ach speck of gold was not worth much, but scooping up handfuls of gold dust made it possible to predict genetic propensities of individuals” (p. 187).

### Polygenic Scores & Their Utility for Sociology

In the past decade, the polygenic score (PGS) approach has emerged as a useful tool in research of complex social traits and behaviors (Belsky and Israel, 2014). A PGS is compound measure that aggregates the genetic effects on a particular outcome. Essentially, once a GWA study estimates the association between all of the genetic variants in the genome and an outcome of interest, these estimates can be summed for individuals, thereby creating individualized compound measure of genetic predisposition. In the following paragraphs, we will discuss four of the most common uses of PGS analysis in social science research, including: (1) genetic mediation, (2) genetic confounding, (3) gene-environment interaction, and (4) gene-environment correlation.

#### Genetic Mediation

One of the most fruitful areas of PGS-based research is the examination of how genetic associations are environmentally mediated. The PGS for educational attainment provides a characteristic example of this kind of analysis. A recent study by Belsky et al. (2018) examined intergenerational mobility in five longitudinal cohorts from three counties. The aim of the study was to test if higher PGSs for educational attainment translated into greater intergenerational mobility (i.e., greater educational and occupational outcomes than one's parents). One of the key findings was that the PGS of mothers predicted their children's educational attainment, even after controlling for the offspring's own PGS. This finding indicates that maternal genes operate through environmental pathways in addition to genetic pathways (i.e., through genetic transmission). Interestingly, environmental mediation has been demonstrated for both transmitted and non-transmitted genetic material (i.e., genetic nurture). For instance, Kong et al. (2018) examined the effects of parental genetic material that was not passed down to their offspring and found that non-transmitted genetic material exerted a genetic effect on educational attainment that was about one third of the size of the child's own PGS. This finding underscores the significant role of environmental mediation in genetic associations as non-transmitted genetic material can only operate through the environmental means.

Phenotypes do not exist in a vacuum: rather, a phenotype like educational attainment is best thought of as being embedded in an interactive network of associations with other phenotypes. Considering the many intermediate and subsequent phenotypes that would result from a genetic propensity for education, studies have shown that a higher PGS for educational attainment also predicts greater social mobility (Belsky et al., 2018; Liu, 2018), labor market earning (Papageorge and Thom, 2018), parental investment (Wertz et al., 2018), and cognitive performance (Lee et al., 2018).

#### Genetic Confounding

A myriad of sociological studies have explored how social factors influence individual behaviors and outcomes. Due to the non-experimental nature of most sociological data, however, selection is a source of constant concern. One important source of selection is human genetics; because roughly all of human behavior is heritable (i.e., influenced by genes; see Polderman et al., 2015), any association between an environmental factor (e.g., delinquent peers) and outcome (e.g., delinquent behavior) *may* be partially attributable to individual's specific genetic markup (Barnes et al., 2014). Advances in modern GWA studies and PGS analysis provide sociologists the opportunity to address some of the unexplained heterogeneity in past research and correct for this kind of selection.

As an example, intergenerational transmission of educational attainment is a central theme in sociological research. One critical challenge in this research is that parents and children share both their living environment and half of their DNA. Because of that, the observed parent-child association in education is both social and genetic. Without considering the genetic influence, an estimate of the socio-environmental influence is likely biased. Using a polygenic score based on the study of Rietveld et al. (2013a), Conley et al. (2015) estimated the genetic confounding effect in the intergenerational transmission of education. They found that genetic factors account for about one-sixth of the observed parent-child association in education. Liu (2018) revisited the issued by using a better powered polygenic score on educational attainment constructed based on the study of Okbay et al. (2016b). His results showed that around one-fifth of intergenerational transmission was accounted for by genetics. These analyses demonstrate not only how genetic contributions to social outcomes can be quantified and controlled for, but also how the inclusion of PGSs can help examine and rule out possible sources of genetic confounding.

#### Gene-Environment Interaction

Arguably one of the most interesting uses of PGS analysis for sociologists is the investigation of gene-environment (G × E) interactions. It is useful to think of G × E interactions as functioning two ways. First, genes may be thought of as moderators of environmental associations, wherein the effects of an environmental exposure are conditional upon an individual's genetic makeup. For example, Guo et al. (2015a) hypothesized that, for college students, the effect of peer influence on binge drinking behavior may vary depending on a student's genetic propensity for alcohol consumption. Using data from the College Roommate Study (ROOM), the authors found that only college students with a moderate level of genetic propensity for consuming alcohol were significantly influenced by a roommate who drank—students of high and low levels of genetic liability were not affected by the influence of peers. While traditional sociological perspectives (e.g., social learning theory) might have predicted a positive and monotonic relationship between peer influence and binge drinking, the inclusion of biologically relevant variables allowed for the identification of a pattern otherwise inexplicable by pure sociological means. In another study, Perry (2016) found evidence for a three-way G × E interaction model, namely higher levels of social integration predicted reduced risk of nicotine dependent among men with high genetic susceptibility to nicotine cravings, yet the protective effect of social integration is substantially reduced among women.

A second way to view G × E interactions is to think of environmental exposures moderating the effects of genetic propensities on later outcomes. Schmitz and Conley (2016), for example, examined the effect of the G × E interaction of a PGS for smoking and Vietnam-era military service on later smoking behavior and health outcomes. Using data from the Health and Retirement Study (HRS), the authors found that veterans (i.e., compared to non-veterans) who possessed PGSs that put them at high risk for tobacco use (i.e., 1–2 standard deviations above the mean) were 57–71% more prone to be smokers later in life and smoke 18–27 more cigarettes per day. Interestingly, rates of smoking later in life did not substantively differ across veterans and non-veterans if they possessed low-medium levels of genetic risk for smoking. This suggests that the environmental exposure of military service only acted as a moderator for individuals who already possessed high genetic predisposition for tobacco use. This again demonstrates how G × E interactions may be employed to more fully explain patterns of unobserved heterogeneity in sociological research.

#### Gene-Environment Correlation

In addition to G × E interactions, gene-environment correlations (*r*GE) has increasingly drawn the attention of social scientists. A *r*GE occurs when an individual's environmental exposure depends on his/her genotype (Plomin et al., 1977; Scarr and McCartney, 1983; Jaffee and Price, 2007; Fletcher and Conley, 2013; Wagner et al., 2013). For instance, Christakis and Fowler (2014) studied the friendship networks of youths in the National Longitudinal Adolescent to Adult Health (Add Health) Study and found a number of significant correlations between members of friend groups at the genetic level. In particular, they found that friends were significantly more homogenous on genes for olfactory function (i.e., their sense of smell) and heterogenous for immune function genes (i.e., compared to random non-friends). This finding provides significant nuance to social network research in that it suggests individuals may sort themselves into groups based on social homophily (e.g., they prefer the same smells) as well as genetic complementarity (e.g., diversity of genetic immune function in groups may help prevent the spread of disease).

Determining the strength of *r*GE is an ongoing area of research. For example, Domingue et al. (2018), also using the Add Health, found that friends showed significant signs of homophily. Friends were more similar (compared to random non-friends in the Add Health) in terms of their genetics for BMI and educational attainment. However, after controlling for genetic similarity at the school level (i.e., keeping comparisons within schools), the overall genome-wide similarity among friends was halved, as was the correlation among friends for the educational attainment PGS (the correlation for BMI was relatively unchanged). This finding suggests that social sources of stratification may be responsible for some amount of *r*GE occurring in society. This study also found that, despite the fact that assortment at the social level accounted for much of the genetic similarity of friends, substantive social-genetic effects still occurred among friend groups. For example, friends' genetics still significantly predicted the educational attainment of group members, even after controlling for genetic similarity.

### Genomic-Relatedness-Matrix Restricted Maximum Likelihood Estimation (GREML)

Genomic-relatedness-matrix restricted maximum likelihood estimation is another innovative approach that handles large-scale genotype data. The basic idea of GREML is to estimate a matrix of genetic relationships among individuals who are not socially related in the sense of being family members. Based on the matrix, the genomic contribution (i.e., collective influence of all genetic variants across the whole genome) can be estimated. This method has also been used to estimate the genomic contribution to human height (Yang et al., 2010), BMI (Yang et al., 2011), schizophrenia (Lee et al., 2012). Social scientists have employed it to investigate the genetic architecture of intelligence (Davies et al., 2011; Chabris et al., 2012), personality traits (Vinkhuyzen et al., 2012), subjective well-being (Rietveld et al., 2013b), and economic and political preferences (Benjamin et al., 2012).

GREML has also been utilized in G × E interaction and *r*GE research. Guo et al. (2015b), for example, conducted a GREML analysis to test if genomic influence on BMI differs by historical period. They found that the genomic influence on BMI was substantially and significantly larger during the obesity epidemic than before in the United States. Using GREML methods, other studies found that the genetic influence was greater among adolescents who lived in adverse social conditions than those in favorable social conditions (Li et al., 2015; Liu et al., 2015). Deary et al. (2012) conducted a bivariate GREML analysis of intelligence at different life stages. As a result, they found a fairly large significant genetic correlation between intelligence in adolescence (age 11) and in late adulthood (age 65–78). In another study, Boardman et al. (2015) estimated genetic correlations among education, BMI, depression, and self-rated health. They showed that observed correlations between education and depression and between education and self-rated health were largely attributable to common genetic factors.

**“*When many such interactions have been defined, the whole can be braided back again to attempt a more complete picture of mental development.”***

-E.O. Wilson, *Consilience* (Wilson, 1999, p. 155–156)

## Future Hurdles and Promising Avenues

This paper has presented an overview of the progress of sociogenomics research, from heritability, and twin studies to candidate genes and finally to the modern era of sociogenomic research, and along the way we have highlighted some of the ways social science researchers have used these techniques to reach new insights. In this final section, we will present future hurdles and promising avenues that we believe are facing sociogenomic research.

### Missing Heritability

Heritability (i.e., the trait variation explainable by genetics) can be estimated from both twin/adoption models and genomic methods; however, family-based trait heritabilities have often dwarfed those estimated from genomic data. For example, family-based heritability of height (a highly biological trait) has been estimated to be as high as 0.80 (i.e., 80% genetic), while SNP heritability estimates have, until recently, only been estimated at less than half that level (Manolio et al., 2009). The discordance between family- and SNP-based estimates has been dubbed the problem of “missing heritability.” The significance of missing heritability for sociogenomics, and genomic research more broadly, is that it signals an incompleteness, either in the method (i.e., GWA and GREML) or in understanding the underlying genetics. A number of possible explanations of missing heritability have been posited, including the inability of GWA analysis to detect the effects of rare genetic variants (i.e., GWA is designed to focus on common variants) or to account for structural variants (i.e., DNA sequences that vary in their number, location, or orientation) (Manolio et al., 2009; see also Eichler et al., 2010).

Although the problem of missing heritability has persisted since the advent of genomic research on human traits and behaviors, recent developments in genomic methods suggest that missing heritability may indeed be recoverable. Wainschtein et al. (2019), using whole-genome data and a variation on the GREML method (discussed above), estimated the heritability of height in a sample of 21,620 unrelated individuals to be 79% (i.e., an estimate consistent with previous family-based estimates). The authors concluded that most of the missing heritability in height was the product of rare variants, which are not detectable using standard genomic data and methods. The ability to assess the contribution of rare variants has begun to close the heritability gap for highly biological traits like height; however, it is possible that different genetic mechanisms are responsible for the missing heritability in more complex social traits such as educational attainment (see Eichler et al., 2010). Time alone will tell if rare variants, or some other heretofore unassessed mechanism, can explain what drives the missing heritability of complex social traits.

### Population Stratification

Although genomic methods have important contributions to make to a wide field of sociological inquiry, current methods are subject to a significant methodological constraint that limits their application to individuals of genetically diverse populations. Modern genomic methodologies (e.g., GWA and PGS studies) are largely limited to individuals with European ancestry due to an evolutionary phenomenon known as population stratification. Population stratification occurs when systematic differences in allele frequencies (i.e., the prevalence of particular versions genes) exist between two subgroups within a larger population. These systematic differences between groups can be used to identify an individual's genetic ancestry; however, if a particular phenotype also breaks along ancestral lines (e.g., chopstick use in Asian countries), population stratification can lead researchers to assume that these systematic differences are causally related to the phenotype itself (e.g., the “chopstick gene”; Hamer and Sirota, 2000).

Due to the potential bias introduced by population stratification, most GWA studies limit their samples to individuals of European descent. Restriction to European populations occurs for, at least, two reasons: (1) genome-wide data on none-European populations is rare (see Popejoy and Fullerton, 2016) and (2) GWA studies are highly dependent on large sample sizes in order to boost the signal of causal SNPs above the genomic noise (for a discussion on GWA studies sample sizes and discovery rates see Visscher et al., 2017). The consequence of these facts is that few GWA studies have assessed non-European samples. Duncan et al. (2018), for example, reported that exclusively European samples were used in 67% of all GWA studies, Asian samples in 19%, and African samples in only 3.8%. The concentration of GWA studies on European samples is especially problematic with regard to PGS construction: a PGS derived from European-based summary statistics may not be reliably applied to individuals from non-European populations.

Duncan et al. (2018) used European-based summary statistics on height, BMI, and schizophrenia to construct PGSs for samples of African and European descent. When comparing the effect sizes, the authors observed that the PGSs for the African ancestry sample were only around 36% as large as the effect sizes for the European sample. Part of the reason European-based PGSs do not function well for individuals of African descent is that their genetic architecture is not wholly overlapping. Sherman et al. (2019) compared the fully sequenced genomes of 910 individuals of African descent to the reference human genome and found that the African genomes possesses around 10% more DNA than does the current human reference genome (the authors did note that the functional significance of these additional regions of DNA are still unknown).

Together, the paucity of non-European based GWA studies and the limited transferability of PGSs to non-European samples means that modern genomic research is (perhaps unavoidably) ignoring numerous segments of the population. These limitations suggest that genomic methodologies may produce a new form of inequality (i.e., inequality in genomic research) between groups of different ancestry in the population (Bustamante et al., 2011; Popejoy and Fullerton, 2016; West et al., 2017). This issue not only has implications for sociological research endeavors that seek to use genomic methods, but also for work being done in fields like medicine and public health (Petrovski and Goldstein, 2016).

### Variance Quantitative Trait Loci

One promising avenue of genomic research for identifying G × E interactions are the current efforts to identify variance quantitative trait loci (vQTLs). As with most regression-based models, GWA studies are designed to discover SNPs that are associated with the mean level of a phenotype; vQTLs, on the contrary, are SNPs that are associated with the variance in a phenotype's expression. Although identifying variance effects across the genome has proven difficult (see Yang et al., 2012; Conley et al., 2018), a recent variance GWA (vGWA) study was conducted using ~350,000 individuals in the UK Biobank that identified 75 genome-wide significant vQTLs associated with variability in body mass index (BMI) (Wang et al., 2019). What is more, these vQTLs were shown to produce significant G × E interactions with five BMI-related environmental factors (i.e., sex, age, physical activity, sedentary behavior, and smoking).

Developments in the area of vQTLs are exciting because they offer researchers the opportunity to examine certain models of interaction that do not readily avail themselves to empirical tests. For instance, researchers have been particularly interested in the differential susceptibility hypothesis of G × E interaction (see Belsky and Pluess, 2009) that states that some individuals are more susceptible to their environment (i.e., doing worse in bad environments and better in supportive ones) compared to others. This model of G × E interaction has large implications because it suggests that improving environmental conditions or relocating individuals to more supportive environments could produce disproportionately positive behavioral responses in some. Yet support for the hypothesis has usually been only partial in nature due to the high level of difficulty in conducting a comprehensive test (i.e., a full test would require an interaction analysis of a plasticity factor [i.e., typically a candidate gene] and an environmental exposure that ranges from highly negative to highly positive). With the advent of vQTL research, however, a convincing test of the differential susceptibility hypothesis that fully leverages modern genomic methods may have finally been made a realistic possibility. vQTL research is still in its infancy, so researchers will need to wait some time until vQTLs for complex traits like educational attainment are identified and made available.

### Phenotypic Annotation

The aim of GWA studies is to estimate the GWA with a specific phenotype; however, the estimates produced by GWA studies represent the average effects of SNPs nested within individuals, all of whom have unique sets of environmental exposures. GWA studies results, and the PGSs derived from them, do not possess information about the mechanistic pathways through which genes exert their influence. This is one area in which sociologists can help improve the state of the research; the rich theoretical traditions of social causation in sociology provide sociogenomics research with a map of environmental associations that are likely to have biological underpinnings, that may mediate the genetic associations, and that could moderate them (Freese, 2018).

The process of mapping the interconnections between associated phenotypes has recently been termed “phenotypic annotation” (Belsky and Harden, 2019), a phrase that mirrors “genotypic annotation” (i.e., the process of mapping biological functions onto sets of specific genes). Phenotypic annotation is one area in which sociologists, in particular, have significant and substantive contributions to make to sociogenomics research. Relying on their wealth of past theory and research, sociologists can inform the phenotypic annotation of socioenvironmental networks that have been established empirically. By using this knowledge, sociogenomics research can proceed to test how far flung genetic effects disperse along the networks of phenotypic associations.

### Epigenetics

Epigenetics is the study of gene regulation (i.e., which genes are read and used for protein synthesis), and one of the most exciting findings to emerge from modern genomics research is that the epigenome is responsive to the environment (e.g., Pembrey et al., 2006). Thus, social scientists have been especially excited to incorporate epigenetics into their work because it provides an opportunity to show how environments might “get under the skin” (Landecker and Panofsky, 2013; Meloni, 2014; Lock, 2015; Harris and McDade, 2018; Meloni et al., 2018). To put it briefly—which will require us to simplify and skip important details (see for more details Deichmann, 2016)—epigenetics is the study of how biological mechanisms can turn genes “on” and “off.” If a gene is turned “on,” it is available to be transcribed and translated, meaning it can have an impact on protein synthesis. If a gene is turned “off,” that gene has essentially been silenced such that it no longer has an impact.

While a thorough discussion of biological mechanisms that determine how genes are regulated is beyond the scope of this paper (but interested readers are encouraged to see National Institute of Health, 2019; see also Szyf and Bick, 2013), what is important for our purposes is that social and environmental factors likely impact the epigenetic process and, thus, it may be possible to identify those relationships through empirical study. For example, imagine two identical twins. On day one of their life, the two twins will be identical in their genome and their epigenome (Fraga et al., 2005). But, as their lives go on and they begin to accumulate unique experiences, their epigenomes will start to diverge. The twin who is exposed to a traumatic event will have certain genes turned on/off that are not affected in the unexposed twin. This is how our environments might get under our skin.

It is probably obvious, at this point, why this area of research has received so much hype (Deichmann, 2016). What may not be as obvious, though, are the theoretical, methodological, and statistical challenges that are involved with epigenetics research. Let us start by recalling one of the complications that has emerged from GWA studies. Specifically, low levels of statistical power exist for identifying any given genetic signal. Now, consider that epigenetic mechanisms work to regulate genes. If the signal from the gene is weak and statistically difficult to identify, then the signal for the epigenome is likely to be even weaker and even more difficult to statistically identify. Epigenetic research thus requires large sample sizes in order to identify reliable signals. Although we are hesitant to say what “large” means, we feel it is safe to say that epigenetics research will likely require sample sizes that are even larger than those required to reliably identify genetic signals with GWA methods.

Nonetheless, there several studies that have drawn on epigenetic data in social science research. One such study was conducted by Beach et al. (2013). Using a sample of *N* = 155 women, these authors found that exposure to childhood sexual abuse was associated with epigenetic change. Moreover, the epigenetic differences appeared to mediate the effect of childhood sexual abuse on later-in-life antisocial behavior. In another example, Lei et al. (2015) relied on a sample of *N* = 99 women and found evidence to suggest that exposure to high-crime neighborhoods had an effect on methylation patterns (i.e., epigenetic markers) of the promoter region of the *5-HTT* candidate gene and that this gene was linked to later depression risk. Taken together, these studies suggest there may be something to the idea that our social environments can indeed get under our skin and impact human behavior in ways that challenge the traditional view that biological and social factors can be considered separate spheres of influence. But low statistical power may be a concern.

Also, more recent evidence complicates this narrative by drawing attention to several practical and methodological challenges. Sugden et al. (2019) uncovered an intricate relationship between epigenetic markers and tobacco smoking. Of course, it has long been recognized that tobacco smoking leads to biological changes, some of which may ultimately result in outcomes such as increased risk of cancer. But there are other, more subtle changes, that occur when one smokes tobacco. Sugden et al. (2019) found evidence that changes in smoking behaviors were linked to changes in epigenetic markers across the human genome. Thus, researchers hoping to study the link between an environmental exposure and epigenetic modifications should consider taking into account the participants' smoking behaviors. If smoking is ignored, it may confound any epigenetic signals that are identified.

Finally, a similar word of caution was expressed by another group of researchers (Marzi et al., 2018) who drew on data from a longitudinal cohort of youth from the United Kingdom (*N* = 2,232). This research team tested for an association between personal victimization experiences and epigenetic markers. They conducted an epigenome-wide analysis (EWA) that attempted to differentiate participants who had experienced victimization and those who had not. If the idea that our environments can become “embedded” and “get under our skin” is right, then we should expect to see epigenetic markers for extremely stressful environmental exposures like victimization. But their analysis failed to identify any epigenetic markers among victims. Marzi et al. (2018, p. 517) concluded with a recommendation that we believe is worth repeating here: “We need to come to terms with the possibility that epigenetic epidemiology is not yet well-matched to experimental, non-human models in uncovering the biological embedding of stress.”

## Conclusion

In his book, *Consilience*, E.O. Wilson envisioned a unification of all branches of knowledge including biology, the humanities, and the social sciences. Likewise, our object in this paper has been the consilience of modern genomic research with the discipline of sociology. Sociogenomics research has experienced its own “jumping together”—the literal meaning of “consilience”—of its two historical approaches perspectives: variance partitioning and mechanism elucidation (Tabery, 2014). Modern genomic methods consist of the construction of variance partitioning polygenic scores that are themselves derived from the mechanism-elucidation process of gene-discovery through GWA studies. In the words of Wilson (1999, p. 12), “[w]e are approaching a new age of synthesis, when the testing of consilience is the greatest of all intellectual challenges.” With the advent and growing robustness of genomic methodologies, sociologists are in an enviable position to adopt these tools and integrate them into their research.

## Author Contributions

HL, JB, and PT designed the paper. PT, RM, RK, JB, and HL wrote the paper.

### Conflict of Interest Statement

The authors declare that the research was conducted in the absence of any commercial or financial relationships that could be construed as a potential conflict of interest.
